# Prof. Hufeland, on the Medical Use of Oil

**Published:** 1801-07

**Authors:** 


### *Prof.* HUFELAND, *on the medical Use of Oil.*

THE action of groſs oils ſeems, in a great meaſure, to proceed chemically, by their affecting organiſation, which, however, will be more ſatisfactorily explained when our knowledge of organic chemiſtry becomes more improved. Oily remedies are found to poſſeſs not only topical effect in mitigating pains, ſpaſms, and other anomalous actions of the nervous ſyſtem, but this effect frequently extends itſelf ſympathetically, by removing, when internally given, ſimilar affections in remote parts. In what manner they properly act, I ſhall leave undetermined, and only acquaint the reader with ſome new caſes, where oils may be ſucceſsfully employed. They have hitherto proved of avail in the following affections.

1ſt.

1. In vehement topical irritations of any part of the inteſtinal canal, theſe remedies are extremely ſerviceable, particularly if thoſe affections are attended with pain, as in cardialgies, colics, diarrhœas, dyſenteries, obſtructio alvi ſpaſtica, and even in the ileus. There is no remedy ſo certain to alleviate almoſt momentarily the moſt vehement colic, as ſome table-ſpoons full of the oil of ſweet almonds, or an emulſion of it, or half an ounce of ſpermaceti diſſolved in warm water. They are at the ſame time very harmleſs, whereas opiates, given in ſimilar inſtances, always require precaution. In febrile infantile diſeaſes they are alſo of great uſe, particularly when, by the frequent uſe of the irritating ſaline remedies, or purgations, a violent fever with a painful and tenſe belly is brought on from a too great irritation of the inteſtinal canal; an oily emulſion will remove theſe ſymptoms.

2d. In all irritations and ſpaſms, general as well as topical, that are occaſioned by worms, oils are the beſt remedies, by diminiſhing the irritation, and preventing inflammation, and by promoting the expulſion of the worms; they are therefore to be much recommended in worm fevers, and likewiſe for ſupporting the effect of anthelmintics, particularly in the cure of the tænia.

They are conſidered as very good remedies in irritations, ſpaſms, and inflammations of the urinary paſſages, as they afford inſtant and certain relief in the moſt vehement pains ariſing from a calculus, in the moſt painful ſpaſms of the bladder, ſtranguries, iſchuries, &c. where either the internal uſe of oils is ſufficient, or at the ſame time the external application in frictions, clyſters, &c.

4th. In the calculus veſicæ felleæ, and the ſymptoms that hence ariſe, as colics, &c. the uſe of oils deſerves much commendation.

5th. They are alſo to be praiſed in ſpaſtic affections of the lungs and breaſt, particularly in the ſpaſmodic aſthma, in the tuſſis ſpaſtica, attended with a ſuppreſſed expectoration and ſome degree of inflammation, both which ſymptoms are generally removed by the uſe of oils. It ought, however, to be obſerved, that if it is too long and unneceſſarily continued, it may occaſion ſuch a relaxation in the lungs that from the immoderate expectoration a phthiſis pituitoſa enſues. The hæmoptyſis ſpaſtica, that ariſes from too great irritability of the lungs, without inflammation, was ſometimes cured by the oil of ſweet almonds, when other remedies proved unſucceſsful.

6th. Oils and fats are the chief remedies in all kinds of poiſoning, particularly that from cauſtic ſubſtances, either externally applied, or given internally. Experience ſhows, that the virulence of arſenical poiſon is remarkably abated, and all danger ger removed, by the internal ufe of oil and milk. Oils prove alfo very great antidotes in the chronic confequences of the poifonous effect of arfenic and lead. The action of animal poifons is likewife diminifhed by the ufe of oil, and the bite of a viper rendered innoxious by rubbing oil on the affected part, which is alfo the beft lenient in the fting of a bee and other infects.

7th. The effects of contagious poifons is even fuppreffed by oils, according to the late obfervations that have been made in the plague, which, in its firft ftage, is cured by repeated inunctions of the whole body with oil. It is probable, that by frictions the fkin is excited to a greater action, and the fpaftic conftriction of the pori cutanei removed by the relaxing quality of the oil, by which means the expulfion of the contagion is promoted: for it has been obferved, that this remedy was only of avail when a great perfpiration enfued.

8th. In all topical pains, fpafms, and conftrictions of external parts, from whatever caufe they may arife, the frictions with oils are the beft and quickeft remedies.

9th. Oil rubbed on the belly has been found very ferviceable in the hydrops afcites, for which purpofe the oil of olives has been particularly employed; but whether this has a proper quality in this cafe, or in what manner it acts, muft be afcertained by farther experiments.

10. In the ancylofis incompleta and ftiffnefs of the limbs, frequent frictions with warm oil are of the greateft fervice.— Thefe are the affections in which oils have been ufed with fuccefs; but I have not met with any inftance mentioned in medical books, of their being employed in the following complaints, in which I experienced their good effect.

11. In vehement dolores poft partum, I ufe the oil of fweet almonds, or of poppy feeds, with the beft fuccefs. Practitioners know to what height thefe pains will rife in fome cafes, how much the neceffary reft of lying-in women is difturbed by them, and how often they give rife to topical inflammations, puerperal fevers, and metaftafes of the milk. Opiates, that are commonly recommended here, may frequently do harm, by either fuppreffing the lochia, or by promoting them too much, and by obftructing the alvine excretions, or by occafioning an inflammatory ftate. All thefe difadvantages are avoided in ufing the oil, by which that fpafm of the uterus is abated, and the neceffary excretions promoted. I generally order one table fpoonful of frefh expreffed oil of fweet almonds to be taken every three hours, either pure or in an emulfion. The firft dofe is frequently fufficient, and there are feldom more than three or four neceffary.

neceſſary. In obſtinate caſes, the addition of one or two grains of extract. hyoſciami to each doſe will improve the effect.

12. In chronic and topical cutaneous diſeaſes, particularly in dry herpetic exanthemata, it is both a ſimple and efficacious remedy, to rub the affected parts with walnut kernels. In ſimilar caſes, the oleum nucis juglandi, proved alſo very uſeful. Several other cutaneous complaints, and even ſcabies, when but topical, were cured in this way.

13. I have frequently employed the oil with ſucceſs in a morbific irritability of the genitals in the male ſex. The irritability of theſe parts is ſometimes ſo much heightened that the moſt trifling ſtimulus occaſions erections, priapiſms, and ſeminal excretions. In this caſe I order the parts to be repeatedly rubbed with oil, of which a little is alſo to be applied between the glans and prepuce; and by thus preventing the debilitating excretions, and anomalous efforts, the proper ſtrength of thoſe parts is re-eſtabliſhed, and the uſe of roborantia greatly ſupported, which are frequently counteracted by that diſagreeable ſymptom.

Oils for medical uſe ought always to be freſh, and expreſſed without heat; whereas, when they are corrupted and rancid, they have a contrary effect, and affect extremely the organs of digeſtion. The moſt efficacious mode is to employ them pure; or, if the ſtomach ſhould not bear them ſo, to give them in an emulſion, viz. ℞. *Olei amygdal. dulcium recenter et frigide expreſs. unc. j. aquæ fontan. unc.* vi. *mucilagin. G. Arabici. q. s. ut F. emulſio, cui adde ſyrup. emulſivi unc. ſs.* D. Although the groſs oils agree with reſpect to their chemical properties, yet they ſeem to differ with regard to the living body; a circumſtance that, moſt probably, depends on the ſubſtances from which they are extracted. The pure freſh expreſſed oil of poppy feeds is the eaſieſt to be digeſted by the ſtomach; and I know inſtances of perſons, whoſe ſtomach could not bear ſallad except what was prepared with the oil of poppy. The oil of lintſeed poſſeſſes the moſt relaxing qualities, to which the oil of ſweet almonds comes the neareſt. The oil of olives is, in ſome meaſure, ſtimulating, and not eaſy of digeſtion. Fats have a more involvent power than pure oils, as they are more compact and gluey, on which account they are more apt to defend a very tender ſurface from the impreſſion of a ſtrong ſtimulus, as of cauſtic poiſons and dyſentery. Amongſt the vegetable fats, the *butyrum de cacao*; and amongſt the animal, the ſpermaceti, are the beſt and eaſieſt of digeſtion. Wax alſo diſſolved in the yolk of eggs, deſerves to be recommended; for, according to ſome authors, this remedy proved of the greateſt avail in violent dyſentries.

